# Maternal Serum Activin A, Inhibin A and Follistatin-Related Proteins across Preeclampsia: Insights into Their Role in Pathogenesis and Prediction

**DOI:** 10.34763/jmotherandchild.20232701.d-23-00002

**Published:** 2023-08-19

**Authors:** Jorge A. Barrero, Laura M. Villamil-Camargo, Jose N. Imaz, Karen Arciniegas-Villa, Jorge A. Rubio-Romero

**Affiliations:** Universidad Nacional de Colombia, Bogotá Campus, Faculty of Medicine, Bogotá, Colombia; Universidad Nacional de Colombia, Bogotá Campus, Faculty of Medicine, Department of Obstetrics and Gynecology, Bogotá, Colombia.

**Keywords:** preeclampsia, inhibins, activins, follistatin

## Abstract

**Background:**

Within the endocrine-paracrine signalling network at the maternal-foetal interface, the activin-inhibin-follistatin system modulates extravillous trophoblast invasion, suggesting a potential role in preeclampsia pathogenesis. This study aimed to compile the evidence published in the last decade regarding the variation in maternal serum activins, inhibin- and follistatin-related proteins in preeclamptic pregnancies compared to healthy pregnancies, and to discuss their role in predicting and understanding the pathophysiology of preeclampsia.

**Material and methods:**

A scoping review was conducted in MEDLINE, EMBASE and LILACS databases to identify studies published within the last ten years (2012–2022).

**Results:**

Thirty studies were included. None of the studies addressed maternal serum changes of isoforms different from activin A, inhibin A, follistatin, and follistatin-like 3. Sixteen studies evaluated the potential of these isoforms in predicting preeclampsia through the area under the curve from a receiver operating characteristic curve.

**Conclusions:**

In preeclampsia, inhibin A is upregulated in all trimesters, whereas activin A increases exclusively in the late second and third trimesters. Serum follistatin levels are reduced in women with preeclampsia during the late second and third trimesters. However, changes in follistatin-like 3 remain inconclusive. Inhibin A and activin A can potentially serve as biomarkers of early-onset preeclampsia based on the outcomes of the receiver operating characteristic curve analysis. Further investigations are encouraged to explore the feasibility of quantifying maternal serum levels of activin A and inhibin A as a clinical tool in early preeclampsia prediction.

## Introduction

Among the great obstetrical syndromes, preeclampsia prevails as one of the leading causes of morbimortality in pregnant women [1], with a global incidence of 5–7%, accounting for more than 70,000 maternal and 500,000 foetal deaths annually [2]. The American College of Obstetricians and Gynecologists [3] defines preeclampsia as a hypertensive pregnancy disorder characterised by a systolic blood pressure ≥140 mmHg and/or diastolic blood pressure ≥90 mmHg in previously normotensive women with more than 20 weeks of gestation. In addition, it must be present with at least one of the following criteria: proteinuria (≥300 mg/24 hours), renal failure (creatinine >1.1 mg/dL, or a two-fold increase in the absence of other renal diseases), thrombocytopenia (<100×10^9^/L), liver dysfunction (ALT/AST≥2 times the upper limit value), pulmonary oedema, acute headache and/or visual disturbances not associated with other causes [3]. Thus, medical evaluation based on the patient's clinical picture remains crucial for a definite diagnosis of preeclampsia; however, early identification of high-risk pregnancies is essential to minimise lethal perinatal outcomes [4].

Accurately predicting preeclampsia in healthy pregnancies remains challenging [5], given its heterogeneous presentation and intricate pathophysiology [6]. In the last decades, quantification of maternal serum biomarkers has emerged as an innovative clinical approach to ensure the surveillance of high-risk pregnancies [7]. Serum measurement of placenta-derived growth factor (PlGF) and soluble fms-like tyrosine kinase-1 (sFlt-1) is currently used as a complementary test to assess the need for early delivery in pregnancies complicated by preeclampsia [8]. Nonetheless, current studies disclose that no predictive test yields an utterly accurate early screening [9], which would otherwise facilitate the initiation of prophylactic therapies [10].

Molecular insights into the common pathogenic pathways involved in preeclampsia onset, such as endothelial dysfunction and disruption of trophoblastic invasion, have highlighted multiple endocrine/paracrine factors that may serve as predictive biomarkers for hypertensive pregnancy disorders [11]. For instance, proteins of the superfamily of the transforming growth factor β (TGF-β) have recently been acknowledged as modulators of the maternal-foetal interface development during the first trimester of gestation [12]. Activins and inhibins, members of the TGF-β superfamily, and follistatin isoforms, their regulatory proteins, have been found ubiquitously expressed in placental tissues and seem to play a crucial role in extravillous trophoblast invasion and spiral arteries remodelling during early placental development [13], suggesting a plausible dysregulation in maternal serum across preeclampsia. Based on the current evidence, this review aims to study variations in maternal serum levels of activins, inhibins, and follistatin-related proteins in preeclamptic pregnancies compared to healthy pregnant women and compile recent studies addressing their predictive role and the molecular mechanisms underlying preeclampsia onset.

## Material and Methods

A scoping review was conducted using the parameters outlined by Arksey and O’Malley [14]. The review aimed to identify studies investigating the variation in maternal serum levels of activin and inhibin isoforms and follistatin-related proteins in pregnant women diagnosed with preeclampsia. A structured search strategy was employed, using the terms (‘Pregnancy’), (‘Preeclampsia’), (‘Activin’), (‘Inhibin’) and (‘Follistatin’), along with their corresponding MeSH and DeCS terms, and truncated Boolean operators (‘AND’ and ‘OR’) in MEDLINE (via PubMed), EMBASE and LILACS databases. Original observational studies with no language restriction and published in peer-reviewed journals between January 2012 and 1 May 2022 were included. The search algorithms used in each database are as follows:

- Medline (PubMed): ((pregnancy) OR (pregnanc*) OR (gestation)) AND ((activins) OR (activin*) OR (fsh-releasing protein*) OR (fsh releasing protein*) OR (‘activin’) OR (inhibins) OR (‘inhibin’) or (inhibin*) or (ovarian inhibin*) OR (female inhibin*) OR (follistatin*) OR (follistatins) or (activin-binding protein*) OR (activin binding protein*)) AND ((preeclampsia) OR (preeclampsia) OR (pre eclampsia) or (pregnancy toxemia*) OR (edema proteinuria hypertension gestosis))
- EMBASE: (pregnancy OR pregnanc* OR gestation) AND (activins OR activin* OR ‘activin’ OR inhibin* OR (ovarian NEAR/3 inhibin*) OR (female NEAR/3 inhibin*) OR follistatin OR follistatin* OR (activin NEAR/5 binding NEAR/5 protein*)) AND (preeclampsia OR (pre AND eclampsia) OR (pregnancy NEAR/3 toxemia*) OR ((edema NEAR/3 proteinuria NEAR/3 hypertension) AND near3 AND gestosis)) AND [2012–2022]/py
- LILACS: ((Embarazo) OR (Gestación)) AND ((Activina) OR (Inhibina) OR (Folistatina)) AND (Preeclampsia)

### Inclusion and exclusion criteria

The initial screening process was carried out based on the following inclusion criteria: (a) non-randomised observational research (case-control and cohort studies) published in the last ten years (2012–2022); (b) studies conducted on a population of pregnant women with singleton pregnancies and diagnosed with any subtype of preeclampsia and; (c) studies that reported the difference in serum levels of activin, inhibin or follistatin isoforms compared to pregnant women without preeclampsia and/or the predictive value of each protein. In addition, to ensure accuracy, literature review studies focused on measuring activin, inhibin, and follistatin isoforms in fluids other than the pregnant women's serum, twin pregnancies and pregnant women with additional comorbidities to preeclampsia were excluded.

### Study screening and selection process

On 1 May 2022, three reviewers (L.M.V.C, J.N.I and K.A.V) conducted a literature search on consulted databases. The titles and abstracts of the retrieved records were compiled and managed using Microsoft Excel 2020. Duplicates were removed in the same program, and two reviewers (J.A.B and J.N.I) independently screened all titles and abstracts to exclude articles irrelevant to the review's objective. Then, studies selected by title and abstract were assessed by full text by four reviewers (J.A.B, L.M.V.C, J.N.I, and K.A.V) to determine their final inclusion based on the previously discussed inclusion criteria. Disagreements in the study selection process were resolved by consensus, and when necessary, an additional reviewer (J.A.R.R) was consulted.

### Data retrieval

For each included study, the following data were extracted: author, year of publication, sample size, protein analysed, weeks of gestation in which the analyte was measured, the subtype of preeclampsia and primary outcomes regarding variation in maternal serum levels. Additionally, depending on the results of each study, the predictive value for preeclampsia of the biomarker studied was extracted, as well as the difference in maternal serum levels of the protein in pregnant women with preeclampsia versus pregnant women with uncomplicated pregnancies. The information was gathered in Table 1.

**Table 1.** Changes in maternal serum activin A, inhibin A, follistatin and FSTL3 in preeclamptic pregnancies.

**Reference**	**Population**	**Protein**	**Weeks of gestation**	**PE subtype**	**Main outcomes**
**Activin A**					
Yu et al. (2012) [15]	Severe PE group: 44 women (30.8±5.1 years) Control group: 51 women (29.8±3.2 years)	Activin A	Five intervals: (1) 15^+0^–18^+0^ (2) 19^+0^–24^+0^ (3) 25^+0^–30^+0^ (4) 31^+0^–35^+0^ (5) 36^+0^–40^+0^	Severe PE	From gestational week 25 until delivery, activin A levels increased significantly in women with severe PE when compared to controls (*p*<0.05). The difference in maternal serum levels of activin A according to week intervals were: (3) 3.6-fold increase in severe PE (16.6 vs. 4.6 ng/mL), (4) 4.2-fold increase in severe PE (30.1 vs.7.2 ng/mL), (5) 3.5-fold increase in severe PE (34.3 vs. 9.7 ng/mL).
Baumann et al. (2013) [16]	PE group: 46 women Controls: 92 women	Activin A	First trimester	No distinction	Activin A increased significantly in pregnancies with PE when compared to controls (*p*<0.05). Activin A yielded an AUC of 0.670 in PE prediction.
Lai et al. (2013) [17]	PE group: 50 women Controls: 250 women	Activin A	Two intervals: (1) 11^+0^–13^+0^ (2) 30^+0^–33^+0^	No distinction	Activin A increased significantly at 30^+0^–33^+0^ weeks of gestation, but not at 11^+0^–13^+0^ weeks, in women with PE (1.47 (1.14–2.38) MoM) when compared to controls (0.99 (0.72–1.42) MoM) (*p*<0.05). Activin A yielded an AUC of 0.722 in PE prediction at 30^+0^–33^+0^ weeks of gestation.
Tarca et al. (2019) [18]	EO-PE group: 33 women (22 (19.0–25.5) years) Controls: 90 women (24 (21.0–27.8) years)	Activin A	22^+1^–28^+0^	EO-PE	Activin A yielded an AUC of 0.890 in EO-PE prediction.
Hao et al. (2020) [19]	PE group: 20 women (31.8±6.0 years) Controls: 20 women (31.9±4.8 years)	Activin A	Five intervals: (1) 5^+0^–9^+0^ (2) 10^+0^–14^+0^ (3) 15^+0^–25^+0^ (4) 26^+0^–33^+0^ (5) 27^+0^–38^+0^	No distinction	Activin A begins to rise around 20 weeks of gestation, yet no significant difference was evidenced between women with PE and normal pregnancies. Activin A yielded an AUC of 0.650 in PE prediction at 16^+0^–30^+0^ weeks of gestation.
Wong et al. (2022) [20]	PE group: 40 women (31.0 [29.3–34.0] years) Control group: 201 women (32.0 [30.0–35.0] years)	Activin A	Two intervals: (1) 27^+0^–29^+0^ (2) 35^+0^–37^+0^	No distinction	Activin A increased significantly at 35^+0^–37^+0^ weeks of gestation, but not at 27^+0^–29^+0^ weeks, in women with PE (706 (2162–8362) pg/mL) when compared to controls (2050 (1018–3723) pg/mL) (*p*<0.001). Activin A yielded an AUC of 0.710 in PE prediction at 35^+0^–37^+0^ weeks of gestation.
**Activin A and inhibin A**					
Li et al. (2016) [43]	PE group: 39 women (27.12±3.12 years) Controls: 100 women (28.67±3.51 years)	Inhibin A Activin A	11^+0^–13^+0^	No distinction	Inhibin A increased significantly in women with PE (1.72±0.02 pg/mL) when compared to controls (1.03±0.06 pg/mL) (*p*<0.001). Activin A increased significantly in women with PE (1.68±0.38 pg/mL) when compared to controls (1.06±0.42 pg/mL) (*p*<0.001).
Xu et al. (2017) [44]	Mild PE group: 14 women (30.8±1.0 years) Severe PE group: 17 women (31.5±1.1 years) Controls: 18 women (30.4±0.7 years) Among the 31 women with PE, 13 had EO-PE and 18 had LO-PE	Inhibin A Activin A	Before delivery. Weeks of gestation were not specified.	Mild PE Severe PE EO-PE LO-PE	Activin A (23.5±2.1 μg/L) increased significantly in women with severe PE when compared to controls and EO-PE (p<0.05). No difference was found with LO-PE. Inhibin A increased significantly in women with severe PE (1.7±0.2 μg/L) and EO-PE (1.9±0.2 μg/L) when compared to controls and mild PE (p<0.001). No difference was found with LO-PE. Both activin A (26.0±2.3 μg/L) and inhibin A (1.9±0.2 μg/L) were significantly higher in EO-PE when compared with women with LO-PE (activin A: 16.3±1.5 μg/L, inhibin A: 1.1±0.1 μg/L) (p<0.001).
**Inhibin A**					
Olsen et al. (2012) [21]	PE group: 459 women, out of which 65 delivered before 34 weeks and 294 delivered after 34 weeks of gestation. Controls: 7,308 women	Inhibin A	Second trimester	EO-PE LO-PE	Elevated inhibin A levels were associated with any form of PE (LO-PE: (OR: 3.08; CI 95%: 2.15–4.42) and EO-PE: (OR: 4.17; CI 95%: 2.11–8.27)). The OR increased for each every >2 MoM rise in inhibin A levels.
Dugoff et al. (2013) [22]	Severe PE group: 26 women PE group: 56 women Controls: 946 women	Inhibin A	11^+0^–14^+0^	Severe PE	Inhibin A increased significantly in pregnancies with PE, but not severe PE, when compared to controls (*p*<0.05).
Boucoiran et al. (2013) [23]	PE group: 34 women (31.5 (26.0–35.0) years) Control group: 584 women (30.0 (27.0–34.0) years)	Inhibin A	Two intervals: (1) 12^+0^–18^+0^ (2) 24^+0^–26^+0^	No distinction	At weeks 12^+0^–18^+0^, inhibin A increased significantly in women with PE (1.10 (0.77–1.52) MoM) when compared to controls (0.96 (0.71–1.25) MoM) (*p*<0.05). In these same weeks, inhibin A yielded an AUC of 0.590 in PE prediction. At weeks 24^+0^–26^+0^, inhibin A increased significantly in women with PE (1.10 (0.89–1.65) MoM) when compared to controls (0.91 (0.66–1.23) MoM) (*p*<0.05). In these same weeks, inhibin A yielded an AUC of 0.620 in PE prediction.
Suri et al. (2013) [24]	PE group: 14 women Controls: 42 women	Inhibin A	15^+0^–22^+0^	No distinction	Inhibin A increased significantly in women with PE (1.37 (0.95–1.62) MoM) when compared to controls (0.96 (0.79–1.33) MoM) (*p*<0.05).
Park et al. (2014) [25]	PE group: 8 women (33 (25–37) years) Controls: 254 women (33 (20–39) years)	Inhibin A	11^+0^–13^+6^	No distinction	Inhibin A increased significantly in pregnancies with PE (1.86 (0.82–2.96) MoM) when compared to controls (1.00 (0.39–3.676) MoM) (*p*<0.05). Inhibin A yielded an AUC of 0.780 in PE prediction.
Giguère et al. (2015) [26]	[1] Preterm PE: Cases: 47 women (29.9±5.5 years) Controls: 94 women (30.3±5.2 years) [2] EO-PE: Cases: 13 women (30.1±6.1 years) Controls: 26 women (30.3±5.4 years) [3] Severe PE: Cases: 68 women (29.7±5.0 years) Controls: 94 women (29.6±4.3 years)	Inhibin A	10^+0^–18^+0^	Preterm PE EO-PE Severe PE	Inhibin A increased significantly in pregnancies with severe PE (267 (176–384) pg/mL) when compared to controls (211 (158–322) pg/mL) (*p*<0.05), and in preterm PE (1.36 (0.97–1.89) MoM) when compared to controls (1.08 (0.85–1.52) MoM). Inhibin A levels in preterm PE and EO-PE did not change significantly when compared to controls.
Kumer et al. (2016) [27]	PE group: 40 women Controls: 28 women	Inhibin A	25^+0^–41^+0^	No distinction	Inhibin A increased significantly in pregnancies with PE (1,267± 383 ng/L) when compared to controls (660 ± 395 ng/L) (*p*<0.001).
Chrelias et al. (2016) [28]	PE group: 98 women (31.1±4.9 years) Control group: 98 women (31.0±5.7 years)	Inhibin A	24 hours before delivery	No distinction	Inhibin A increased significantly in women with PE (1.07±0.83 ng/mL) when compared to controls (0.73±0.35 ng/mL) (*p*<0.05). PE was highly associated with changes in inhibin A levels (OR: 1.09). The likelihood of developing PE increased by 10% for every rise in inhibin A serum levels by 0.1 ng/mL.
Broumand et al. (2018) [29]	PE group: 14 women Controls: 286 women	Inhibin A	16^+0^–20^+0^	No distinction	The sensitivity of inhibin A levels ≥1.25 MoM in PE prediction was 83.83%, its specificity 65.30%, positive predictive value 4.67%, negative predictive value 99.48%, positive likelihood 2.41, and negative likelihood 0.24. Its diagnostic accuracy was 65.66%. Inhibin A levels were directly associated with the incidence of PE (*p*<0.001) and its severity (*p*<0.05).
Belovic et al. (2019) [30]	104 pregnant women (30.54±4.93 years), of which 6 developed PE. No available data of the control group.	Inhibin A	16^+0^–19^+0^	No distinction	No significant difference was evidenced in inhibin A levels among women with PE (1.23±0.11 MoM) and normal pregnancies (1.17±0.98 MoM).
Yue et al. (2020) [31]	PE group: 560 women (29.3±2.72 years) Controls: 8333 women (29.0±2.82 years)	Inhibin A	14^+0^–20^+0^	No distinction	Inhibin A increased significantly in pregnancies with PE (1.34±0.83 MoM) when compared to controls (1.07±0.47 MoM) (*p*<0.001). Inhibin A was found to be an independent risk factor for preeclampsia (OR: 7.45; CI 95%: 4.69–11.85).
Sharabi-Nov et al. (2020) [32]	PE group: 31 women (33.9 (32.3–35.6) years) Controls: 21 women (34.0 (32.0–35.9) years)	Inhibin A	>24^+0^and before delivery	No distinction	Inhibin A increased significantly in pregnancies with PE (2,097 (1,546–2,660) pg/mL) when compared to controls (724 (491–904) pg/mL) (*p*<0.05).
Kim et al. (2021) [33]	PE group: 13 women (33.50±6.80 years) Controls: 338 women (33.90±4.0 years)	Inhibin A	14^+0^–22^+0^	No distinction	No significant difference was evidenced in inhibin A levels among women with PE (1.00 (0.39–2.29) MoM) and normal pregnancies (0.93 (0.06–3.86) MoM).
Keikkala et al. (2021) [34]	EO-PE: 11 women (31.8±4.6 years) LO-PE: 34 women (31.2±5.4 years) Controls: 89 women (31.8±5.1 years)	Inhibin A	Three intervals: (1) 12^+0^–14^+0^ (2) 18^+0^–20^+0^ (3) 26^+0^–28^+0^	EO-PE LO-PE	Inhibin A increased significantly in pregnancies with PE at weeks 18^+0^–20^+0^ (*p*<0.05) and 26^+0^–28^+0^ (*p*<0.001) when compared to controls. At weeks 26^+0^–28^+0^, inhibin A yielded an AUC of 0.717 in PE prediction, AUC of 0.889 in EO-PE prediction, and AUC of 0.674 in LO-PE prediction.
**Follistatin and activin A**					
Charkiewicz et al. (2018) [36]	Mild PE group: 21 women (29±5.65 years) Controls: 27 women (28±5.49 years)	Follistatin Activin A	25^+0^–40^+0^	Mild PE	No significant difference was evidenced in activin A levels among women with PE (801.40±729.47 pg/mL) and normal pregnancies (260.28±212.90 pg/mL). Follistatin decreased significantly in women with PE (20,753.30 ± 3,196.87 pg/mL) when compared to controls (33,290.16±3,217.63 pg/mL) (*p*<0.05).
**Follistatin-related proteins**					
Han et al. (2014) [40]	PE group: 39 women (30.54±4.06 years), of which 10 had mild PE and 29 had severe PE. Controls: 73 women (29.10±3.40 years).	FSTL3	24^+0^–28^+0^	Mild PE Severe PE	FSTL3 increased significantly in women with PE (26.68±8.20 ng/ml) when compared to controls (21.93±9.02 ng/ml) (*p*<0.001). No significant difference was evidenced in FSTL3 levels among women with mild PE (29.10±10.29 ng/mL) and severe PE (25.84±7.54 ng/mL).
Garcés et al. (2015) [35]	PE group: 20 women (19.5 (18–26) years) Controls: 28 women (23.5 (19–30) years)	Follistatin	Three periods of gestation: (1) 11^+5^–12^+5^ (2) 24^+2^–24^+6^ (3) 34^+2^–35^+4^	No distinction	Follistatin decreased significantly during late pregnancy (34^+2^–35^+4^ weeks of gestation) in women with PE (116.2±50.1 ng/mL) when compared to controls (151.2±36.2) (*p*<0.05).
Horvath et al. (2016) [42]	PE group: 32 women Controls: 44 women	FSTL3	Third trimester Other gestational periods were not specified.	No distinction	FSTL3 increased significantly during the third trimester in PE pregnancies (25.30 ng/mL) when compared to controls (15.64 ng/mL) (*p*<0.001) but did not in earlier gestational time periods.
Charkiewicz (2016) [37]	Mild PE group: 12 women Controls: 12 women	Follistatin	25^+0^–40^+0^	Mild PE	Follistatin decreased significantly in women with mild PE when compared to controls (*p*<0.05). Follistatin yielded an AUC of 0.771 in mild PE prediction.
Luo and Han (2017) [39]	PE group: 33 women (30.50±4.12 years) Controls: 71 women (29.30±3.35 years)	FSTL3	24^+0^–28^+0^	No distinction	FSTL3 increased significantly in women with PE (0.55 (0.00–1.34) MoM) when compared to controls (1.21 (0.58–1.97) MoM) (*p*<0.05). FSTL3 yielded an AUC of 0.706 in PE prediction.
Nevalainen et al. (2017) [38]	EO-PE group: 29 women in the training set (28 (26–33) years) and 42 women in the test set (29 (25–33) years) Controls: 652 women in the training set (30 (26–34) years) and 141 women in the test set (29 (25–33) years)	FSTL3	9^+0^–13^+6^	EO-PE	No significant difference was evidenced in FSTL3 levels among women with PE and normal pregnancies in both sets. FSTL3 yielded an AUC of 0.750 in the training set, and an AUC of 0.650 in EO-PE prediction.
Purut et al. (2019) [41]	PE group: 13 women Controls: 131 women	FSTL3	11^+0^–14^+0^	No distinction	No significant difference was evidenced in FSTL3 levels among women with PE (10.85±5.55 pg/mL) and normal pregnancies (11.25±4.86 pg/mL). FSTL3 yielded an AUC of 0.522 in PE prediction.

Abbreviations: FSTL3: Follistatin-like 3, PE: Preeclampsia, EO PE: Early-onset PE, LO PE: Late-onset preeclampsia, MoM: Multiple of the median, AUC: Area under the curve, OR: odds ratio.

## Results

**Figure 1.**
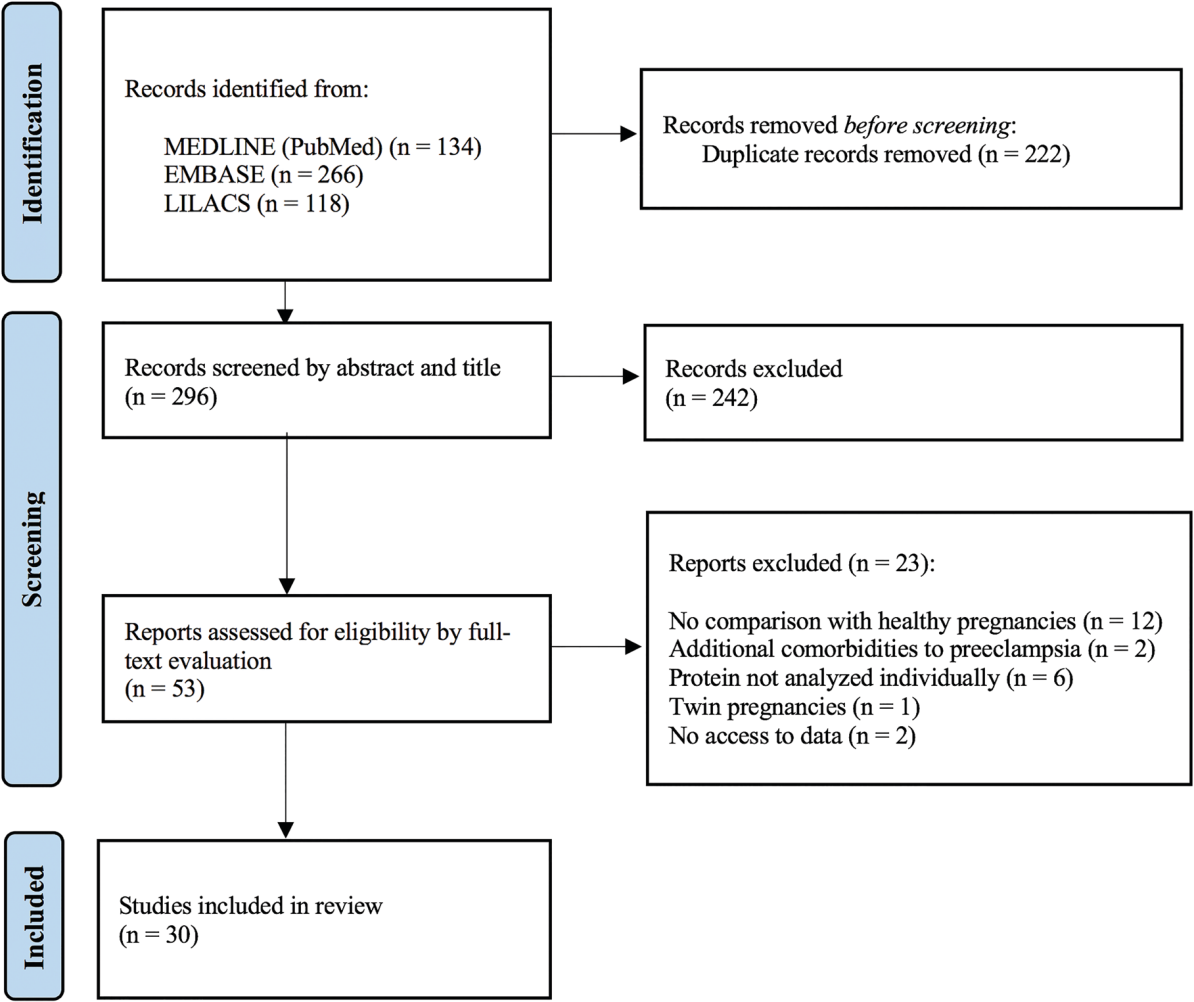
Flow diagram of study selection.

A total of 518 studies were identified from the initial search in MEDLINE (via PubMed), EMBASE and LILACS databases. After removing duplicates and screening studies based on title and abstract, 53 studies were evaluated in full text. At last, 30 studies aligned with the review scope and were included. The overall selection process is depicted in Figure 1, and the characteristics of the selected studies are presented in Table 1. The records gathered in the present review display the findings of the investigations into the activin-inhibin-follistatin system in preeclampsia in the last ten years. Table 1 shows that only activin A, inhibin A, follistatin, and follistatin-like 3 were studied concerning maternal serum changes in preeclampsia. From the total number of papers retrieved, six studies evaluated changes in activin A levels [15,16,17,18,19,20], and fourteen assessed inhibin A [21,22,23,24,25,26,27,28,29,30,31,32,33,34]. Only three studies investigated maternal serum variation of follistatin exclusively [35,36,37], while five focused on follistatin-like 3 [38,39,40,41,42]. Two studies assessed both inhibin A and activin A in parallel [43,44], and only one addressed both follistatin and activin A simultaneously [36].

Multiple subtypes of preeclampsia were reported, including early onset preeclampsia (<34 weeks of gestation) [18,21,26,34], late-onset preeclampsia (≥34 weeks of gestation) [21,34], severe preeclampsia as defined by the International Society for the Study of Hypertension in Pregnancy [15,22,26], and preterm preeclampsia [26], which required delivery before 37 weeks. Sixteen studies addressed the role of activin A, inhibin A, or follistatin-related proteins in preeclampsia risk and prediction through the estimation of the odds ratio (OR) [21,28,31] and area under the curve (AUC) from a receiver operating characteristic (ROC) curve [16,17,18,19,20,23,25,29,34,37,38,39,41]. However, only one study [29] evaluated the positive and negative predictive value of inhibin A, and none assessed these parameters for activin A or follistatin-related proteins.

## Discussion

The role of placental endocrine-paracrine signalling is pivotal in the instauration of the maternal-foetal interface suited to meet the metabolic demand of the conceptus throughout gestation. When this signalling system is disrupted in states of placental stress, an imbalance in growth factors secretion occurs and results in systemic vascular dysfunction often associated with preeclampsia pathogenesis [2]. The interaction between the decidua and the blastocyst trophoblast cells is one of the earliest events in placental development, and it allows the extravillous trophoblast to invade the endometrium lining setting in proximity to the maternal spiral arteries [45]. Thus, gene expression regulation throughout trophectoderm differentiation is crucial for proper placentation and appears to be tightly modulated by multiple endocrine/paracrine factors [12]. As such, recent evidence has shown the importance of the activin-inhibin-follistatin system in the maternal-foetal interface signalling during the early stages of differentiation, migration and invasion of the extravillous trophoblast [13].

### Insights into the activin-inhibin-follistatin system

Inhibins are heterodimeric glycoproteins made up of α and β subunits that assemble two main isoforms: inhibin A (α-βA) and inhibin B (α-βB). While these hormones are mainly expressed by the granulosa cells upon luteinizing hormone and follicle-stimulating hormone influx, from the fourth week of gestation, inhibin A is predominantly secreted by the fetoplacental unit [46] rather than by ovarian follicles or the corpus luteum [47]. On the other hand, activins are homodimeric glycoproteins with three isolated and classified isoforms according to the combination of β subunits: activin A (βAβA), activin AB (βAβB) and activin B (βBβB). Activin A is the most predominant isoform in humans [48] and exhibits an increase in maternal serum throughout gestation that reflects the fetoplacental unit secretion [49]. Interestingly, within the maternal-foetal interface, activins elicit multiple functions in a paracrine, endocrine and autocrine fashion that hinders its classification solely as a classic hormone [48].

While not recognised as hormones per se, follistatin-related proteins are still tightly involved in multiple signalling pathways in the female reproductive system [50]. Follistatin is a glycoprotein encoded by the *FST* gene, which following alternative splicing, expresses a series of follistatin-related proteins that bind to peptides of the TGF-β superfamily, thereby inhibiting ligand-receptor complex formation [51]. Similar to both inhibin A and activin A, placental follistatin secretion is responsible for the rise in serum levels during pregnancy [52]. Investigations in animal models have shown that follistatin might be involved in decidualisation [53] and ovarian suppression throughout gestation [54].

**Figure 2.**
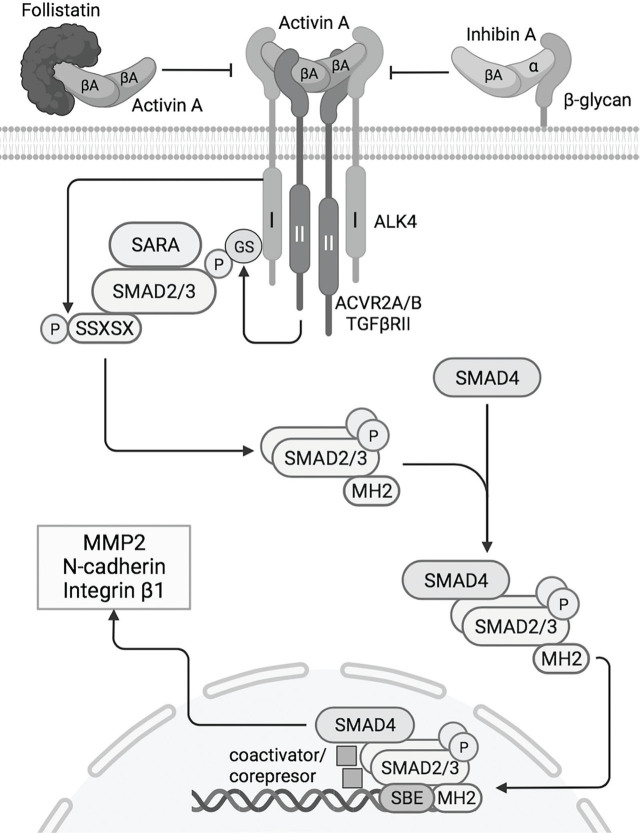
Activin A canonical signalling pathway in the maternal-foetal interface. Activin binding enables receptor activation by acquiring a tetrameric conformation through ALK4 (TβR-I) interaction with one of the following TβR-II: ACVR2A, ACVR2B or TGFβRII [55]. Activated TβR-II phosphorylates the GS domains of ALK4, which then gain high affinity for the receptor-regulated SMAD proteins (SMAD2 and SMAD3) [56]. Facilitated by the auxiliary adaptor protein SARA, SMAD2 and SMAD3 are recruited in proximity to the kinase domains of ALK4 and, upon binding, they are phosphorylated on the SSXSX motifs [87]. Ser/Thr phosphorylation of receptor-SMADs induces dimerisation and exposure of the nuclear translocation region on their MH2 domain and augments their affinity for a Co-SMAD (SMAD-4) [87]. SMAD2/3-SMAD2/3-SMAD4 heterotrimers are then translocated to the nucleus and bind to specific DNA regions (SBE sequence) and transcriptional coactivators or corepressors [88]. Inhibin A binds to TβR-II through its βA subunit but blocks the phosphorylation of ALK4 by its α subunit, which binds to extracellular matrix β-glycans and block receptor tetramerization [88]. Follistatin-related proteins block ligand-receptor complex formation by binding activins. Abbreviations: TβR-I, type I subunit receptor; TβR-II, type II subunit receptor; ALK4, activin-like kinase 4; ACVR2A/B, activin receptor type 2A/B; TGFβRII, transforming growth factor-β receptor type 2; MMP2, matrix metalloproteinase 2. Source: own elaboration.

Activins exert their functions through binding to heterooligomeric transmembrane receptors with intrinsic Ser/Thr kinase activity made up of two type I subunits and two type II subunits [55]. Signal transduction and transcriptional response are mediated by the Sma- and Mad-related proteins (SMADs), particularly SMAD2 and SMAD3 [56]. In contrast, inhibin A and follistatin-related proteins downregulate activin A signalling by distinct mechanisms. A more detailed explanation of the canonical signalling pathways regulated by activin A, inhibin A, follistatin and follistatin-like 3 in the maternal-foetal interface is presented in Figure 2. The activin-inhibin-follistatin system plays a crucial physiological role in normal placentation and may also serve as a predictive biomarker. Hence, hypotheses about their role in preeclampsia pathogenesis are discussed below based on the variation in maternal serum across preeclamptic pregnancies.

### Activin A

During the first trimester of gestation, activin A is expressed by the syncytiotrophoblast, cytotrophoblast and decidual cells [57,58]. The type II subunit receptor ALK4 is expressed in the extravillous trophoblast and holds the highest affinity for activin A [59], indicating the presence of paracrine signalling within the maternal-foetal interface. Nonetheless, activin A reaches the systemic circulation, as evidenced by Silver et al. [60], who found that elevated levels of activin A in pregnancy result more accurately from increased placental secretion rather than higher levels of inhibin A. As illustrated in Figure 2, the transcriptional response to activin A augments the expression of N-cadherin [61], integrin β1 [62] and matrix metalloproteinase 2 (MMP2) via canonical ALK4-SMAD2/3 pathways [59]. In normal placentation, N-cadherin allows extravillous trophoblast adhesion, thereby conferring the proliferative, migratory and invasiveness capacity [63] aided by MMP2, which promotes decidual stromal invasion by extracellular matrix degradation and regulation of vascular remodelling [64]. More recently, integrin β1 has been recognised to enhance extravillous trophoblast migration and invasion coupled with reduced cellular apoptosis via PI3K and Akt signalling pathways [65].

Based on the evidence gathered in this review, in preeclamptic pregnancies activin A levels rise significantly across the third and late second trimesters, from 25 weeks of gestation [15], between 30^+0^–37^+0^ weeks [17,20] and until delivery [44]. However, two studies found that while levels increased, there was no significant difference with healthy pregnancies [19,36]. In contrast, changes in activin A during the first trimester are inconclusive, as recent evidence shows both a substantial increase in preeclampsia [16] between 11^+0^–13^+0^ weeks of gestation [43] and no significant difference in early gestational periods (11^+0^–25^+0^ weeks) [15,17,19]. While activin A stimulates trophoblast invasiveness [13], its upregulation in early or late gestation as a possible pathogenic mechanism in preeclampsia onset has been explored to a lesser extent.

A recent study conducted by Brkić et al. [66] revealed that overexpression of SMAD2, a canonical second messenger activated by activin A through ALK4 signalling, significantly suppressed the ability of trophoblasts to form endothelial-like networks conveying a potential link between elevated activin A levels and preeclampsia pathogenesis. Furthermore, as activin A is secreted into the systemic circulation, it plays an essential role in preeclampsia pathophysiology by prompting endothelial oxidative stress [67]. The underlying mechanism lies in SMAD2/3 phosphorylation, which increases the production of NADPH oxidase 2 (NOX2), a primary source of reactive oxygen species in the endothelial cells resulting in endothelial dysfunction [67]. Moreover, NOX2 expression seems inversely proportional to endothelial nitric oxide synthase (eNOS) expression; hence, indirect decrease of eNOS due to activin A elevation reduces vasodilator stimuli, favouring the onset of hypertension [67].

Other hypotheses suggest that activin A involvement in preeclampsia pathogenesis rely on endothelin-1, ICAM-1 and VCAM-1 overexpression, favouring hypertension and oedema [68]. Furthermore, antepartum activin A elevation has been directly associated with abnormal longitudinal strain, indicating myocardial dysfunction in women with hypertensive pregnancy disorders [69]. Interestingly, recent evidence suggests that prophylactic aspirin administration reduces maternal serum activin A, significantly improving global longitudinal strain and decreasing postpartum cardiac dysfunction in preeclamptic women [70]. Results from these studies suggest that elevated activin A levels might be one of the factors underlying preeclampsia pathogenesis and further cardiovascular risk later in life.

Maternal activin A levels were evaluated for their ability to predict preeclampsia in all trimesters of gestation. In the ROC curve analysis, activin A yielded an AUC that appeared to increase according to gestational weeks: in the first trimester an AUC of 0.670 was reported [16], between 16^+0^–30^+0^ weeks the AUC was 0.710 [19], and between 30^+0^–33^+0^ weeks an AUC of 0.722 was reported [17]. In addition, one study assessed maternal activin A levels near delivery (35^+0^–37^+0^ weeks), and an AUC of 0.710 was obtained [20]. Although no subtype of preeclampsia was clearly defined in the studies mentioned before, one study demonstrated that maternal serum activin A yielded an AUC of 0.890 in early-onset preeclampsia prediction at 22^+1^–28^+0^ weeks of gestation. As such, maternal activin A could convey a potential complementary biomarker for preeclampsia prediction, particularly early-onset preeclampsia based on the obtained AUC value, when measured in the second and third trimesters. However, determining its predictive value with greater accuracy requires future investigation.

### Inhibin A

Inhibin A is expressed in both decidua [71] and trophoblast-derived tissues: syncytiotrophoblast [72] and cytotrophoblast [73]. During pregnancy, placental secretions are the primary source of maternal serum inhibin A [74], and as shown by Taché et al. [75], pregnancies with elevated inhibin A levels exhibit a higher risk of developing early-onset (RR: 5.0) and late-onset (RR: 2.3) severe preeclampsia. Supporting this evidence, the studies gathered in this review reported an increase of inhibin A in maternal serum levels between 10^+0^–22^+0^ weeks [22,24,25,26,31,34], and from week 25^+0^ until delivery [27,28,32,34] in pregnancies complicated with preeclampsia. While 12 out of 14 investigations disclose elevated inhibin A levels in maternal serum, Belovic et al. [30] and Kim et al. [33] reported no statistically significant difference between women who developed preeclampsia and those with healthy pregnancies. Overall, maternal inhibin A levels seem to be significantly augmented across the first, second and third trimester in preeclampsia.

As such, early elevations in inhibin A levels interfere with placentation in response to activin A signalling downregulation [76] (Figure 2), while later elevations are associated with an accelerated cytotrophoblast differentiation that results in villous cytotrophoblast depletion potentially leading to preeclampsia onset [77]. Disease pathogenesis and systemic manifestations in preeclamptic women could also be prompted by inhibin A disruption of maternal blood vessels diminishing the adaptability of the cardiovascular system to pregnancy [31] and reduction of placental irrigation aggravating placental ischemia [31]. Inhibin A is associated with placental hypoxia, an important trigger in preeclampsia pathogenesis, as shown by Depoix et al. [78] who found that hypoxia-inducible factors and endothelial PAS domain protein 1 (EPAS1) upregulate inhibin α subunit in human trophoblast cells.

Furthermore, inhibin A has been widely investigated as a possible biomarker for preeclampsia prediction due to its inclusion in non-invasive Down syndrome screening [79]. In the ROC analysis for preeclampsia prediction, inhibin A yielded an AUC of 0.780 at 11^+0^–13^+6^ weeks [25], an AUC of 0.590 at 12^+0^–18^+0^ weeks and 0.620 at 24^+0^–26^+0^ weeks [23]. Between 26^+0^–28^+0^ weeks of gestation, Keikkala et al. [34] showed an overall AUC of 0.717 for preeclampsia prediction, and when addressing preeclampsia subtypes, they found an AUC of 0.889 and 0.674 in early-onset preeclampsia and late-onset preeclampsia prediction, respectively. Notably, current guidelines advise preeclampsia prophylaxis with aspirin to be initiated before 16 weeks of gestation [80]. Hence, inhibin A elevation between 11^+0^–13^+6^ weeks [25], unlike activin A, could aid in the early identification of at-risk women, potentially enabling timely prophylactic therapy initiation.

The elevation of inhibin A in maternal serum has been identified as an independent risk factor for preeclampsia (OR: 7.45; CI 95%: 4.69–11.85) [31] and appeared to be associated both with late-onset preeclampsia (OR: 3.08; CI 95%: 2.15–4.42) and early-onset preeclampsia (OR: 4.17; CI 95%: 2.11–8.27) [21]. The likelihood of preeclampsia was also found to increase by 10% for every 0.1 ng/mL rise in maternal serum inhibin A [28]. Broumand et al. [29] assessed the predictive value of inhibin A levels ≥1.25 MoM in preeclampsia prediction at 16^+0^–20^+0^ weeks of gestation. They reported a sensitivity of 83.83%, a positive predictive value of 4.67%, a negative predictive value of 99.48%, a positive likelihood of 2.41, a negative probability of 0.24 and a diagnostic accuracy of 65.66%. As such, the authors stated that inhibin A levels were directly associated with the incidence of preeclampsia and its severity [29].

### Follistatin and follistatin-like 3

Within the family of follistatin-like proteins, follistatin-like 3 exhibits the highest homology to follistatin, differing only in length sequence and the presence of heparin-binding and Kazal-like domains [51]. In healthy pregnancies, follistatin levels increase significantly in all trimesters compared to non-pregnant women [54]. Additionally, placental tissues have shown an upregulated expression of follistatin-related proteins and β-glycans that serve as binding proteins for activins and inhibins, respectively [81] (Figure 2). According to the results derived from this study, follistatin decreases between the gestational weeks 25^+0^–40^+0^ [36,37] and 34^+2^–35^+4^ weeks of gestation [35], while follistatin-like 3 showed an increase in the late second (24^+0^–28^+0^ weeks) [39,40] and third trimester [42] but no significant changes were evidenced between 9^+0^–14^+0^ weeks [38,41].

Recent evidence shows that follistatin elicits a significant inhibitory effect on both trophoblast invasion and activin A secretion at 10^+0^–12^+0^ weeks of gestation [73], though the results of Nevalainen et al. [38] and Purut et al. [41] show no significant increase in preeclampsia within this period. The downregulation of TGF-β signalling by follistatin-related proteins, particularly activin A inhibition by follistatin and follistatin-like 3, requires a 2:1 ratio between the binding protein and the corresponding ligand [82]. Hence, in preeclamptic women, a reduction of follistatin-related proteins during the second and third trimester is observed, resulting in an increase in free activin A, leading to oxidative stress and endothelial dysfunction [82]. Placentation dysfunction mediated by follistatin-like 3 upregulation [83] has shown to be associated not only with activin A inhibition [84,85] but also with binding of GDF8/myostatin, which enhances extravillous trophoblast cells invasiveness [86]. In addition, follistatin-like 3 regulates trophoblast proliferation, apoptosis and lipid metabolism, suggesting that late elevations in follistatin-like 3 will favour pathogenic processes related to placental dysfunction; however, the signalling pathways involved remain uncertain [83].

Only one study has evaluated follistatin variation in mild preeclampsia prediction in the late second and third trimesters in the past decade, with an AUC of 0.771 when measured between the 25^+0^–40^+0^ weeks of gestation [37]. Follistatin-like 3, on the other hand, yielded lower values in the ROC analysis for preeclampsia prediction; AUC of 0.650–0.750 between 9^+0^–13^+6^ weeks of gestation [38], an AUC of 0.522 between 11^+0^–14^+0^ weeks of gestation [41] and an AUC of 0.706 between 24^+0^–28^+0^ weeks of gestation [39]. Follistatin-related proteins exhibit significant variation in maternal serum across pregnancies, though based on the results of this review, a substantial difference between preeclamptic and uncomplicated gestations is evident from the late second trimester. As such, early prediction might be hindered given the late upregulation and downregulation of follistatin-like 3 and follistatin, respectively. Moreover, the scarcity of longitudinal studies assessing their predictive value limits their utility as a biomarker, and further investigations are required to establish their role in preeclampsia prediction accurately.

### Limitations

As the studies included in this review evaluated different subtypes of preeclampsia, the heterogeneity of the population in each investigation undermines the generalisability of the results regarding the change in maternal serum levels of activin A, inhibin A and follistatin-related proteins in preeclampsia. In addition, although weeks of gestation are indicated in most studies, the wide intervals limit the accuracy in determining the period in which a significant change in maternal serum levels of these proteins occurs. Therefore, further studies with greater homogeneity and narrower intervals of weeks of gestation are recommended. Nevertheless, the results of this review allow us to estimate the variation of maternal serum activin A, inhibin A and follistatin-related proteins in preeclamptic pregnancies.

## Conclusion

In the last decade, assessments of maternal serum levels of the activin-inhibin-follistatin system proteins disclose both subtle and significant changes across preeclampsia. An upregulation of inhibin A has been consistently documented in all trimesters. Increased activin A was reported in the late second and third trimesters compared to uncomplicated pregnancies. Moreover, serum follistatin levels in women with preeclampsia are significantly reduced in the late second and third trimesters. However, changes in follistatin-like 3 are still inconclusive, as evidenced by upregulation and no significant change.

Although fewer studies have investigated their predictive value, the findings presented in this review suggest that inhibin A and activin A hold a potential utility as biomarkers of early-onset preeclampsia, given that they display the highest AUC values in this subtype. Follistatin and follistatin-like 3 showed lower predictive potential than activin A and inhibin A based on the outcomes of the ROC curve analysis. We encourage further longitudinal investigations into the clinical application of these biomarkers and studies to elucidate the variation of activin A, inhibin A and follistatin-related proteins in preeclampsia as a manifestation of a maternal disease or as markers of abnormal placentation.

### Key points

- Within the maternal-foetal interface, activin A signalling via ALK4-SMAD2/3 pathways enhances extravillous trophoblast migration and reduces cellular apoptosis. While its upregulation appears to disrupt trophoblasts’ endothelial-like network formation, its role in preeclampsia pathogenesis remains the subject of future research.
- Elevation of inhibin A in maternal serum throughout preeclamptic pregnancies suggests a disturbance in normal placentation, given its role in activin A signalling downregulation by α subunit binding and inhibition of receptor tetramerisation.
- Maternal serum inhibin A is significantly upregulated from 10^+0^ week of gestation in preeclampsia compared to healthy pregnant women. This could aid in the early identification of at-risk women and allow for the timely initiation of aspirin prophylactic therapy.
- Follistatin serum levels in women with preeclampsia are significantly reduced at the late second and third trimesters (25^+0^–40^+0^ weeks of gestation), though changes in maternal serum follistatin-like 3 remain uncertain.
- Based on the yielded AUC values from the ROC curve analysis, maternal serum activin A and inhibin A might serve as predictive biomarkers of early-onset preeclampsia; before 34^+0^ weeks of gestation.
